# Weekend admission and mortality for gastrointestinal disorders across England and Wales

**DOI:** 10.1002/bjs.10608

**Published:** 2017-09-19

**Authors:** S E Roberts, T H Brown, K Thorne, R A Lyons, A Akbari, D J Napier, J L Brown, J G Williams

**Affiliations:** Swansea University Medical School, Swansea University, Swansea, UK; Farr Institute of Health Informatics Research, Swansea University, Swansea, UK; Department of Gastroenterology, Gloucestershire Royal Hospital, Gloucester, UK

## Abstract

**Background:**

Little has been reported on mortality following admissions at weekends for many gastrointestinal (GI) disorders. The aim was to establish whether GI disorders are susceptible to increased mortality following unscheduled admission on weekends compared with weekdays.

**Methods:**

Record linkage was undertaken of national administrative inpatient and mortality data for people in England and Wales who were hospitalized as an emergency for one of 19 major GI disorders.

**Results:**

The study included 2 254 701 people in England and 155 464 in Wales. For 11 general surgical and medical GI disorders there were little, or no, significant weekend effects on mortality at 30 days in either country. There were large consistent weekend effects in both countries for severe liver disease (England: 26·2 (95 per cent c.i. 21·1 to 31·6) per cent; Wales: 32·0 (12·4 to 55·1 per cent) and GI cancer (England: 21·8 (19·1 to 24·5) per cent; Wales: 25·0 (15·0 to 35·9) per cent), which were lower in patients managed by surgeons. Admission rates were lower at weekends than on weekdays, most strongly for severe liver disease (by 43·3 per cent in England and 51·4 per cent in Wales) and GI cancer (by 44·6 and 52·8 per cent respectively). Both mortality and the weekend mortality effect for GI cancer were lower for patients managed by surgeons.

**Discussion:**

There is little, or no, evidence of a weekend mortality effect for most major general surgical or medical GI disorders, but large weekend effects for GI cancer and severe liver disease. Lower admission rates at weekends indicate more severe cases. The findings for severe liver disease may suggest a lack of specialist hepatological resources. For cancers, reduced availability of end-of-life care in the community at weekends may be the cause.

## Teaser

Worst for gastrointestinal cancer and liver disease

## Introduction

A weekend effect of increased mortality for admissions on weekends, compared with normal weekdays, has been investigated extensively in recent years^1–3^. Large weekend effects greater than 14 per cent have been reported previously for particularly high-risk acute conditions including stroke^3–6^, subarachnoid haemorrhage^3,7^, abdominal aortic aneurysm^3,8,9^ and pulmonary embolism^3,9–11^, as well as various cancers^9,12^.

Gastrointestinal (GI) diseases are the leading cause of hospital admission and the third leading cause of death in the UK after circulatory and respiratory diseases^13^. However, other than several reports on the weekend effect for upper GI bleeding^3,14–21^, and two reports from Australia^12^ and Canada^9^ on the weekend effect for some GI cancers, little has been reported for other GI conditions.

The primary objective of this study was to establish whether a wide range of major GI disorders are susceptible to the so-called weekend effect on mortality, following unscheduled hospital admission. Further objectives were to determine whether any weekend effect varies according to the type of GI disorder (general surgical or medical, hepatic and cancer), and also to establish whether there are any differences in patient or clinical factors between weekday and weekend admissions. Importantly, to provide confirmatory evidence, the study was designed to use data collected independently from the two separate national health services in England and Wales.

## Methods

### Study population

The study covered emergency admissions among adults (aged at least 18 years) for major GI disorders to all public hospitals across England and Wales from 1 January 2004 to 31 December 2012. These were identified from national administrative inpatient data, Hospital Episode Statistics for England (population 53·5 million in 2012) and the corresponding Patient Episode Database for Wales (population 3·07 million). The inpatient data were linked systematically to mortality data from the Office for National Statistics and the Welsh Demographic Service to identify all deaths that occurred after discharge from hospital, as well as inpatient deaths, within 30 days of admission. These information sources have been used extensively by the present authors for previous studies of mortality following hospitalization for GI and other disorders^3,15,22–24^. The information sources were compiled, stored and accessed through a secure, privacy-protected, data storage gateway, the Secure Anonymised Information Linkage (SAIL) databank^25–27^, supported by the Farr Institute of Health Informatics Research. The ascertainment of mortality and the record linkage methodology, based on a unique, anonymized, encrypted linking field for each patient, have been validated as greater than 98 per cent and more than 99·8 per cent accurate^26^.

Using methodology described previously^24^, each person's first emergency admission following the start of the study period was included, and then also subsequent admissions provided they occurred at least 30 days after discharge from a preceding emergency admission.

Ethical approval for the study data was not required as it is based on anonymized data. Approval was obtained instead from the independent Information Governance Review Panel, which includes members of the National Health Service (NHS) National Research Ethics Service, British Medical Association, Caldicott Guardians, Public Health Wales NHS Trust, NHS Wales Informatics Service and members of the public.

### Gastrointestinal disorders

The study covered all ‘major’ GI disorders that carry substantial mortality. These were defined by a principal ICD-10 diagnostic category at discharge that led to at least 50 deaths in England and Wales within 30 days of acute hospitalization during the study interval. Nineteen ICD-10 categories relating to GI disorders fulfilled these criteria. These were designated as ‘general surgical or medical GI disorders’ (11 conditions), ‘hepatic disorders’ (2) and ‘GI malignancy’ (6).

The 11 general surgical or medical GI disorders were: upper GI bleeding, perforated peptic ulcer and peritonitis, gastritis, non-hiatal hernia, inflammatory bowel disease, non-infective gastroenteritis, intestinal obstruction, diverticular disease, gallstone disease, acute pancreatitis and intestinal infections.

The hepatic disorders were alcoholic liver disease and hepatic failure, which were also grouped together as ‘severe liver disease’, as they are the most life-threatening forms of liver disease (ICD-10 codes are shown in *Appendix S1*, supporting information). Additionally, alcoholic liver disease was differentiated according to the following aetiologies, which each led to more than 50 deaths at 30 days in England and Wales: alcoholic hepatitis, alcoholic cirrhosis of liver and alcoholic hepatic failure. GI malignancies were assessed overall and by major cancers (oesophageal, gastric, colorectal, liver, pancreatic and gallbladder).

### Study outcome and ‘exposure’ measures

The main outcome measure was the weekend effect, which was defined as the percentage increased or decreased mortality (at 30 days after acute admission) for admissions on weekends compared with admissions on weekdays. Weekends were defined as 00.00 hours on Saturday to 23.59 hours on Sunday. Public holidays were not counted as weekend or weekday admissions, and were excluded from the analysis of weekend effects. The secondary outcome measure was mortality at 30 days, established using the numbers of admissions for each GI disorder as denominators and the numbers of deaths (conventionally from all causes) as numerators.

A possible weekend effect for severe liver disease was assessed according to whether or not patients were admitted to one of the six hospitals in England in which liver transplant centres were located and, for patients admitted, according to whether they were local patients. The transplant centres were located throughout the study period in Birmingham, Cambridge, Leeds, London (2) and Newcastle^28^, and local patients were defined as resident in local authorities served by the trusts in which the transplant centres were located.

When assessing GI cancers, upper GI bleeding and non-hiatal hernias, the weekend effect was compared according to whether or not the patients came under the care of a surgical consultant (recorded during either the first or last episodes of the admission spell). Similarly for hepatic disorders, the weekend effect was assessed according to whether or not the patients were managed by a consultant gastroenterologist or hepatologist.

### Statistical analysis

Methods of analysis included multivariable logistic regression modelling to adjust for the weekend effect (mortality odds ratios for weekend *versus* weekday admissions), patient age (in 5-year groups from age 35 to more than 85 years, with age less than 35 years as the reference category), sex and 11 major patient co-morbidities (ischaemic heart disease, other cardiovascular disease, cerebrovascular disease, other circulatory disease, malignancy, liver disease, chronic obstructive pulmonary disease (COPD), asthma, diabetes, renal failure and dementia; ICD-10 codes are listed for each co-morbidity in *Appendix S1*, supporting information). Co-morbidities were based on a diagnosis recorded in any position on the patient's current inpatient record, or on previous inpatient records during the preceding 5 years. In the multivariable modelling, to eliminate any possible bias in the determination of patient co-morbidity from inpatient admissions alone, adjustment was also made for patients with no admissions during the preceding 5 years. In a further analysis of weekend effects, additional adjustment was made for social deprivation quintile^29,30^, year of admission, whether or not the patients were managed surgically, the admission source (accident and emergency department, general practitioner, consultant clinic or other source), day of death (weekend or weekday) and hospital size (England: fewer than 200, 200–399, 400–599, 600–799, 800–999 and 1000 or more beds; Wales (with relatively fewer hospitals): fewer than 200, 300–399, 400–599 and 600 or more beds).

Relative survival was calculated as a ratio to compare the observed survival in the hospitalized patients with that expected in the corresponding (age- and sex-matched) general populations of England and Wales, and was presented graphically up to 30 days after admission. The expected mortality was calculated by applying age- and sex-specific mortality rates in the general population with the corresponding numbers of study patients in each quinquennial age and sex stratum^23,24^.

Other methods of analysis included mortality rates with associated 95 per cent confidence intervals, calculated using the exact method; admission rates; Mann–Whitney *U* tests to compare median length of stay for patients admitted on weekdays and weekends; and *t* tests to compare mean patient ages and numbers of co-morbidities. Mortality rates were calculated using the number of deaths at 30 days as numerator and the number of admissions as denominator, and are expressed as percentages. Admission rates were calculated using the number of study patients as numerator, the resident populations of England and Wales (based on the mid-study year, 2008) as denominator, and are expressed per 100 000 population. The Bonferroni correction was applied to adjust for multiple testing of disorders, although this correction can be regarded as conservative. Significance was measured at the conventional 5 per cent level; all tests were two-tailed. The methodology reported follows the STROBE statement^31^ and RECORD (REporting of studies Conducted using Observational Routinely collected Data) guidelines (http://www.record-statement.org/). The analysis software used was SPSS® version 22 (IBM, Armonk, New York, USA).

## Results

For the 19 emergency GI disorders included in the study, a total of 2 254 701 patients were admitted in England and 155 464 in Wales. The mean(s.d.) age of the patients was 60·7(20·2) years in England and 59·8(20·1) years in Wales. A majority of the patients (53·3 per cent in England and 53·5 per cent in Wales) were women.

Patient age and sex were missing in less than 0·01 per cent of cases (England: 153 and 5 respectively of the 2 254 701 admissions; Wales: 1 and 5 of the 155 464 admissions). Residential local authority was missing for 21 470 (1·0 per cent) of patients in England and 377 (0·2 per cent) in Wales, and social deprivation in 29 662 (1·3 per cent) and 3603 (2·3 per cent) respectively. Residential local authority was also missing for 3·5 per cent of patients (145 of 4170) admitted to liver transplant centres. Consultant specialty was missing for 0·03 per cent (786) of patients in England and for no patients in Wales. With little or no influence on the study findings, missing data were excluded from the analyses involving the respective factors. There were no missing data for day of admission and death, year of admission, or hospital size.

### Comparison of patient and clinical factors for weekday and weekend admissions

For each GI disorder, *Table 1* shows the number of admissions, population admission rates, median lengths of stay, mean ages and numbers of co-morbidities for patients admitted at the weekend and on weekdays. For the 19 GI disorders overall, admission rates were 27·5 per cent lower at weekends than on weekdays in England and 34·4 per cent lower in Wales, with the greatest reduction in weekend admissions for GI cancer (by 44·6 per cent in England and 52·8 per cent in Wales) and for severe liver disease (43·3 per cent in England and 51·4 per cent in Wales). Patients admitted at the weekend had a similar number of recorded co-morbidities to those admitted on weekdays (for the 19 GI disorders combined: mean 1·6 *versus* 1·6 in England (*P* = 0·701) and 1·7 *versus* 1·7 in Wales (*P* = 0·452)). They were also of similar age (mean 60·5 *versus* 60·7 years in England and 61·0 *versus* 61·7 years in Wales) and had a similar duration of inpatient stay (median 4·0 days for both groups in both England and Wales), although, with the large study sizes, the slight differences were significant (*P* < 0·001). For each GI disorder, admission at the weekend, compared with on weekdays, was proportionately more often through accident and emergency departments rather than via general practitioners or consultant clinics (*Table 2*). For most disorders, the proportion of patients managed surgically was similar on weekdays and at weekends (*Table 2*).

**Table 1:** Comparison of patients admitted on weekdays and at weekends for major emergency gastrointestinal disorders according to number of admissions, population admission rate, duration of inpatient stay, patient age and co-morbidities, in England and Wales

	No. of admissions		Median length of stay (days)^*^		Mean patient age (years)		Mean no. of co-morbidities	
	Weekdays	Weekends	Weekdays	Weekends	Weekdays	Weekends	Weekdays	Weekends
England								
General surgical and medical GI disorders								
Upper GI bleeding	172 364 (66·1)	55 670 (53·3)	7	6	56·1	55·4	1·9	1·8
Perforated peptic ulcer and peritonitis	31 047 (11·9)	10 048 (9·6)	9	9	62·2	61·7	1·8	1·8
Gastritis	89 684 (34·4)	28 552 (27·4)	1	1	54·0	52·6	1·5	1·4
Non-hiatal hernia	100 921 (38·7)	25 694 (24·6)	2	3	63·1	64·4	1·6	1·6
Inflammatory bowel disease	82 067 (31·5)	17 379 (16·7)	6	5	43·8	43·8	0·9	0·9
Non-infective gastroenteritis	235 079 (90·1)	80 254 (76·9)	2	2	59·5	59·4	1·8	1·7
Intestinal obstruction	103 554 (39·7)	33 440 (32·0)	6	6	67·3	67·5	1·9	1·9
Diverticular disease	120 937 (46·3)	34 629 (33·2)	5	4	67·9	68·8	1·8	1·9
Gallstone disease	268 265 (102·8)	85 808 (82·2)	4	4	58·1	57·8	1·4	1·3
Acute pancreatitis	92 510 (35·5)	33 457 (32·1)	5	5	54·9	55·2	1·5	1·5
Intestinal infection	120 955 (46·5)	41 949 (40·2)	4	3	60·6	60·2	1·8	1·8
Severe liver disease								
Alcoholic liver disease	71 392 (27·4)	15 748 (15·1)	9	9	51·6	51·4	1·7	1·8
Alcoholic hepatitis	10 646 (4·1)	2297 (2·2)	10	9	46·7	46·7	1·3	1·3
Alcoholic cirrhosis of liver	21 609 (8·3)	4858 (4·7)	9	9	54·0	53·5	2·1	2·2
Alcoholic liver failure	8522 (3·3)	1981 (1·9)	12·5	12	52·4	52·6	1·3	1·3
Hepatic failure	7964 (3·1)	2264 (2·2)	7	6	56·8	56·6	2·1	2·1
GI cancer								
Overall	214 617 (82·3)	47 543 (45·6)	10	11	71·4	71·4	1·5	1·5
Oesophageal	36 130 (13·8)	7722 (7·4)	7	7	71·2	71·2	1·4	1·5
Gastric	27 315 (10·5)	6013 (5·8)	8	9	72·8	72·6	1·4	1·5
Colorectal	88 635 (34·0)	20 726 (19·9)	11	12	71·5	71·7	1·5	1·5
Liver	14 585 (5·6)	3095 (3·0)	11	10	72·0	69·6	2·0	2·1
Pancreatic	33 988 (13·0)	7039 (6·7)	11	10	71·3	71·0	1·5	1·6
Gallbladder	2325 (0·9)	449 (0·4)	13	12	72·2	71·6	1·5	1·5
Wales								
General surgical and medical GI disorders								
Upper GI bleeding	10 522 (68·9)	3080 (50·4)	7	6	56·3	54·0	1·9	1·9
Perforated peptic ulcer and peritonitis	1753 (11·5)	559 (9·1)	10	10	64·3	64·2	2·0	1·8
Gastritis	6755 (44·2)	2011 (32·9)	2	2	54·4	52·0	1·5	1·4
Non-hiatal hernia	7199 (47·1)	1718 (28·1)	2	3	63·2	65·4	1·7	1·8
Inflammatory bowel disease	4574 (29·9)	945 (15·5)	6	5	45·9	46·5	1·1	1·1
Non-infective gastroenteritis	16 395 (107·0)	4941 (80·8)	3	3	60·7	60·4	1·9	1·8
Intestinal obstruction	6177 (40·4)	1934 (31·6)	6	5	67·7	67·9	2·0	1·9
Diverticular disease	9419 (61·6)	2510 (41·1)	5	4	68·1	68·8	1·9	2·0
Gallstone disease	22 137 (144·9)	6759 (110·6)	4	4	58·2	57·6	1·4	1·3
Acute pancreatitis	5612 (36·7)	1896 (31·0)	6	5	56·6	56·8	1·5	1·5
Intestinal infection	7113 (46·5)	2247 (36·8)	4	4	62·0	61·0	2·0	2·0
Severe liver disease								
Alcoholic liver disease	5658 (37·0)	1074 (17·6)	9	9	52·3	51·9	1·6	1·6
Alcoholic hepatitis	574 (3·8)	95 (1·6)	10	10·5	47·3	47·0	1·1	1·2
Alcoholic cirrhosis of liver	1711 (11·2)	299 (4·9)	9	8	54·4	53·8	1·9	1·8
Alcoholic liver failure	473 (3·1)	120 (2·0)	11·5	11·5	52·4	53·1	1·7	1·8
Hepatic failure	514 (3·4)	126 (2·1)	8	5·5	58·8	57·4	2·0	1·9
GI cancer								
Overall	16 666 (109·1)	3144 (51·4)	12	11	72·3	72·0	1·6	1·6
Oesophageal	2769 (18·1)	479 (7·8)	8	6	71·3	71·6	1·5	1·5
Gastric	2351 (15·4)	404 (6·6)	10	11	73·5	72·8	1·5	1·5
Colorectal	6954 (45·5)	1447 (23·7)	13	12	72·8	72·1	1·6	1·5
Liver	1001 (6·6)	182 (3·0)	13	10·5	71·6	71·4	2·0	2·0
Pancreatic	2531 (16·6)	444 (7·3)	11	11	71·7	71·9	1·6	1·5
Gallbladder	151 (1·0)	31 (0·5)	14·5	12	73·3	75·8	1·5	1·6

Values in parentheses show admission rate per 100 000 population.

* Excludes patients who died within 30 days of admission. GI, gastrointestinal.

**Table 2:** Comparison of patients admitted on weekdays and at weekends for major emergency gastrointestinal disorders according to source of admission and surgical management, in England and Wales

	Admitted via A&E (%)		Admitted via GP (%)		Admitted via consultant clinic (%)		Managed surgically (%)	
	Weekday	Weekend	Weekday	Weekend	Weekday	Weekend	Weekday	Weekend
England								
General surgical and medical GI disorders								
Upper GI bleeding	68·8	83·7	25·5	12·9	0·8	0·1	16·4	18·3
Perforated peptic ulcer and peritonitis	68·5	78·4	19·4	12·9	3·7	0·7	76·4	77·4
Gastritis	68·7	82·9	24·1	13·1	1·2	0·1	38·5	41·6
Non-hiatal hernia	55·7	70·6	36·9	24·2	1·3	0·2	96·5	96·2
Inflammatory bowel disease	51·4	73·4	26·8	19·6	11·0	1·1	34·4	40·5
Non-infective gastroenteritis	63·6	75·2	27·5	18·1	1·9	0·2	29·5	29·8
Intestinal obstruction	62·8	72·4	29·4	22·2	2·0	0·2	87·5	88·5
Diverticular disease	59·7	74·0	33·5	21·7	1·5	0·2	87·2	87·7
Gallstone disease	67·4	78·5	26·1	17·3	1·3	0·1	83·9	85·4
Acute pancreatitis	78·2	85·2	17·4	11·6	0·7	0·1	91·1	92·1
Intestinal infection	66·1	75·4	26·6	18·8	1·3	0·2	20·4	20·6
Severe liver disease								
Alcoholic liver disease	60·7	82·7	28·0	12·1	3·4	0·4	9·7	12·1
Alcoholic hepatitis	60·6	80·2	30·9	13·5	2·3	0·5	10·8	13·6
Alcoholic cirrhosis of liver	60·1	80·2	26·5	11·7	4·2	0·5	10·4	12·7
Alcoholic liver failure	60·6	82·2	30·9	13·5	2·3	0·5	9·4	11·4
Hepatic failure	63·0	78·7	25·1	14·6	3·4	0·3	14·0	14·9
GI cancer								
Overall	41·1	61·1	30·8	22·1	10·9	1·4	41·4	42·1
Oesophageal	37·8	59·4	29·9	22·9	12·2	1·3	23·9	21·1
Gastric	41·9	61·6	30·1	20·2	10·4	1·3	28·6	27·4
Colorectal	42·7	61·2	30·5	21·2	10·5	1·4	57·1	59·0
Liver	42·4	62·6	29·4	19·8	10·4	1·9	28·2	28·9
Pancreatic	29·9	59·7	34·1	24·6	10·6	1·4	32·8	31·4
Gallbladder	39·8	62·6	31·4	19·8	9·9	1·6	44·1	45·7
Wales								
General surgical and medical GI disorders								
Upper GI bleeding	52·2	72·5	44·6	25·5	0·8	0·0	16·9	17·1
Perforated peptic ulcer and peritonitis	58·0	69·6	32·6	25·6	3·9	2·0	75·4	80·9
Gastritis	50·2	68·3	40·6	28·6	1·1	0·0	39·8	42·0
Non-hiatal hernia	42·6	58·3	54·1	40·1	1·1	0·1	96·7	96·8
Inflammatory bowel disease	37·5	56·7	49·4	41·4	8·5	0·5	43·5	50·6
Non-infective gastroenteritis	43·2	57·3	51·4	37·3	1·6	0·3	25·9	23·3
Intestinal obstruction	47·3	56·5	47·0	41·1	2·4	0·2	86·1	88·5
Diverticular disease	44·0	59·1	42·9	33·0	1·0	0·1	89·3	91·0
Gallstone disease	55·0	66·7	42·1	31·8	0·8	0·1	85·8	87·0
Acute pancreatitis	65·1	76·8	32·9	22·0	0·5	0·1	93·0	93·7
Intestinal infection	43·9	55·2	50·6	40·9	1·4	0·1	16·7	16·1
Severe liver disease								
Alcoholic liver disease	44·5	73·6	49·2	24·4	3·3	0·1	5·7	7·7
Alcoholic hepatitis	44·3	72·6	52·8	25·3	1·0	0·0	4·9	10·5
Alcoholic cirrhosis of liver	44·7	73·2	49·0	24·1	3·0	0·0	6·1	8·7
Alcoholic liver failure	49·9	76·7	46·7	21·7	1·7	0·0	6·3	6·7
Hepatic failure	44·7	68·3	47·7	26·2	4·7	0·8	10·1	7·9
GI cancer								
Overall	29·3	50·8	50·7	40·3	8·3	0·8	45·1	47·9
Oesophageal	27·3	49·5	49·7	40·3	10·9	1·5	32·1	29·4
Gastric	29·3	52·8	47·8	43·6	7·4	0·7	31·9	35·1
Colorectal	30·8	48·4	52·5	37·7	7·6	0·7	57·4	63·2
Liver	30·3	50·0	53·0	40·7	8·6	1·1	32·3	30·8
Pancreatic	27·2	49·5	55·6	45·3	7·8	0·5	41·4	38·5
Gallbladder	29·8	54·8	53·6	38·7	9·9	0·0	50·1	64·5

A&E, accident and emergency department; GP, general practitioner; GI, gastrointestinal.

### Comparison of 30-day mortality for weekday and weekend admissions

#### General surgical and medical gastrointestinal disorders

For the 11 general surgical and medical GI disorders, there was little or no evidence of significant weekend admission effects on mortality in England or Wales (*Table 3*). However, after correction for multiple testing across the 11 disorders, there were significant weekend effects in England for two disorders: upper GI bleeding (9·9 (95 per cent c.i. 6·1 to 14·0) per cent; *P* < 0·001) and non-hiatal hernia (27·7 (18·6 to 37·6) per cent; *P* < 0·001). In patients managed surgically, there was no weekend effect for upper GI bleeding (4·1 (−4·3 to 13·1) per cent) and a slightly reduced weekend effect for non-hiatal hernia (25·0 (15·5 to 35·1) per cent).

**Table 3:** Mortality at 30 days and percentage increased mortality after emergency admission for major gastrointestinal disorders at weekends compared with weekdays, in England and Wales

	No. of admissions	No. of deaths at 30 days	Crude mortality rate (%)	Increased mortality for weekend admissions (%)^*^
England				
General surgical and medical GI disorders				
Upper GI bleeding	232 762	17 822	7·7 (7·6, 7·8)	9·9 (6·1, 14·0)
Perforated peptic ulcer and peritonitis	42 303	9458	22·4 (22·0, 22·8)	8·0 (1·7, 14·6)
Gastritis	120 675	1279	1·1 (1·0, 1·1)	−5·9 (−17·9, 7·8)
Non-hiatal hernia	128 755	4376	3·4 (3·3, 3·5)	27·7 (18·6, 37·6)
Inflammatory bowel disease	101 042	980	1·0 (0·9, 1·0)	14·0 (−3·3, 34·3)
Non-infective gastroenteritis	322 727	10 261	3·2 (3·1, 3·2)	3·9 (−0·8, 8·8)
Intestinal obstruction	139 882	15 388	11·0 (10·8, 11·2)	7·8 (3·5, 12·3)
Diverticular disease	158 390	6201	3·9 (3·8, 4·0)	11·7 (5·1, 18·8)
Gallstone disease	361 616	4408	1·2 (1·2, 1·3)	−0·2 (−7·2, 7·3)
Acute pancreatitis	128 885	5317	4·1 (4·0, 4·2)	5·9 (−1·0, 13·2)
Intestinal infection	166 662	7314	4·4 (4·3, 4·5)	−5·4 (−10·6, 0·1)
Severe liver disease				
Alcoholic liver disease	88 589	15 752	17·8 (17·5, 18·0)	27·4 (21·8, 33·3)
Alcoholic hepatitis	13 178	1524	11·6 (11·0, 12·1)	7·6 (−7·0, 24·4)
Alcoholic cirrhosis of liver	26 929	4966	18·4 (18·0, 18·9)	25·3 (16·0, 36·3)
Alcoholic liver failure	10 686	3689	34·5 (33·6, 35·4)	19·8 (10·9, 29·3)
Hepatic failure	10 435	2852	27·3 (26·5, 28·2)	14·5 (2·4, 28·0)
GI cancer				
Overall	266 340	74 991	28·2 (28·0, 28·3)	21·8 (19·1, 24·5)
Oesophageal	44 574	12 978	29·1 (28·7, 29·5)	29·4 (22·7, 36·4)
Gastric	33 861	10 251	30·3 (29·8, 30·8)	26·2 (18·9, 34·1)
Colorectal	111 162	25 543	23·0 (22·7, 23·2)	18·5 (14·3, 22·8)
Liver	17 915	6547	36·5 (35·8, 37·3)	25·0 (15·2, 35·5)
Pancreatic	41 646	15 406	37·0 (36·5, 37·5)	28·8 (22·0, 35·8)
Gallbladder	2820	1012	35·9 (34·1, 37·7)	21·9 (−1·5, 50·9)
Wales				
General surgical and medical GI disorders				
Upper GI bleeding	13 861	1034	7·5 (7·0, 7·9)	−3·5 (−17·7, 13·2)
Perforated peptic ulcer and peritonitis	2365	600	25·4 (23·6, 27·2)	−0·3 (−22·2, 27·7)
Gastritis	8926	79	0·9 (0·7, 1·1)	−2·1 (−44·3, 72·0)
Non-hiatal hernia	9213	322	3·5 (3·1, 3·9)	13·2 (−14·4, 49·9)
Inflammatory bowel disease	5598	56	1·0 (0·8, 1·3)	−23·0 (−64·5, 67·2)
Non-infective gastroenteritis	21 811	635	2·9 (2·7, 3·1)	15·0 (−4·8, 38·9)
Intestinal obstruction	8264	968	11·7 (11·0, 12·4)	2·5 (−13·3, 21·1)
Diverticular disease	12 171	394	3·2 (2·9, 3·6)	13·1 (−11·8, 45·0)
Gallstone disease	29 498	346	1·2 (1·1, 1·3)	23·1 (−4·2, 58·3)
Acute pancreatitis	7686	373	4·9 (4·4, 5·4)	3·3 (−20·1, 33·7)
Intestinal infection	9561	444	4·6 (4·2, 5·1)	−11·8 (−30·9, 12·5)
Severe liver disease				
Alcoholic liver disease	6821	1153	16·9 (16·0, 17·8)	26·2 (6·1, 50·2)
Alcoholic hepatitis	676	97	14·3 (11·8, 17·2)	−33·9 (−68·8, 40·2)
Alcoholic cirrhosis of liver	2039	375	18·4 (16·7, 20·1)	41·0 (2·9, 93·3)
Alcoholic liver failure	603	235	39·0 (35·1, 43·0)	53·1 (−2·7, 141·0)
Hepatic failure	652	184	28·2 (24·8, 31·9)	57·3 (−1·5, 151·0)
GI cancer				
Overall	20 121	5748	28·6 (27·9, 29·2)	25·0 (15·0, 35·9)
Oesophageal	3291	951	28·9 (27·4, 30·5)	34·2 (8·8, 65·6)
Gastric	2798	890	31·8 (30·1, 33·6)	39·7 (11·7, 74·8)
Colorectal	8532	2010	23·6 (22·7, 24·5)	26·0 (10·4, 43·9)
Liver	1197	459	38·3 (35·6, 41·2)	36·3 (−2·0, 89·4)
Pancreatic	3034	1100	36·3 (34·5, 38·0)	20·0 (−3·0, 48·6)
Gallbladder	185	72	38·9 (31·9, 46·4)	−33·0 (−73·1, 66·7)

Values in parentheses are 95 per cent confidence intervals.

* These weekend mortality effect sizes are adjusted for patient age, sex and 11 major co-morbidities (for ICD-10 codes, see *Appendix S1*, supporting information) and are based on odds ratios from logistic regression modelling. GI, gastrointestinal.

#### Severe liver disease

There were large and significant weekend effects for alcoholic liver disease (England: 27·4 per cent, *P* < 0·001; Wales: 26·2 per cent, *P* = 0·009), and also for hepatic failure in England (14·5 per cent; *P* = 0·019). In Wales, the weekend effect for hepatic failure was greater than in England, but marginally non-significant (57·3 (95 per cent c.i. −1·5 to 151·0) per cent; *P* = 0·060) (*Table 3*). When differentiating the different aetiologies of alcoholic liver disease, there were large weekend effects for alcoholic liver failure and alcoholic cirrhosis of the liver, but not for alcoholic hepatitis (*Table 3*). When alcoholic liver disease and hepatic failure were combined as ‘severe liver disease’, the weekend increased mortality effects were 26·2 (21·1 to 31·6) per cent in England and 32·0 (12·4 to 55·1) per cent in Wales.

In both England and Wales, overall mortality for severe liver disease was significantly lower among patients managed by a consultant hepatologist or gastroenterologist (*Table 4*). Mortality was also significantly lower among patients treated at one of the six specialist liver transplant centres in England, although most of this reduction was for people who were not local residents (*Table 4*). The weekend mortality effect for severe liver disease was also substantially lower among patients admitted to a liver transplant centre (49·4 per cent reduction) or managed by a consultant hepatologist or gastroenterologist (45·1 per cent lower in England and 24·2 per cent lower in Wales), although these differences were marginally non-significant (*Table 4*).

**Table 4:** Mortality at 30 days and percentage increased mortality after emergency admission at weekends, compared with weekdays, for gastrointestinal conditions according to admission source and management, in England and Wales

Management and admission source	No. of admissions	Crude 30-day mortality (%)	Increased mortality for weekend admissions (%)^*^
England			
GI cancer			
Management			
Surgical specialty	112 190	20·9 (20·7, 21·2)	14·0 (9·8, 18·4)
All other specialties	154 150	33·4 (33·2, 33·7)	27·7 (24·3, 31·3)
Admission source			
A&E	119 182	31·2 (30·9, 31·4)	12·5 (9·3, 15·3)
GP	77 602	31·5 (31·2, 31·8)	27·9 (22·4, 33·6)
Consultant clinic	24 069	17·6 (17·2, 18·1)	−22·6 (−37·1, −2·3)
Other	44 857	20·4 (20·1, 20·8)	9·8 (2·2, 16·9)
Severe liver disease			
Management			
Consultant hepatologist or gastroenterologist	37 470	16·0 (15·7, 16·4)	16·7 (8·3, 25·7)
All other specialties	61 554	20·5 (20·2, 20·8)	30·4 (23·9, 37·2)
Admitted to			
Liver transplant centre	4170	12·9 (11·9, 14·0)	13·6 (−10·0, 43·6)
Resident in same local authority^†^	2268	15·4 (13·9, 16·9)	20·0 (−11·0, 61·7)
Resident in other local authority^†^	1757	9·7 (8·4, 11·2)	18·1 (−21·2, 77·2)
All other transplant centres	94 854	19·0 (18·8, 19·3)	26·9 (21·6, 32·4)
Admission source			
A&E	64 519	20·0 (19·7, 20·3)	16·4 (11·0, 22·1)
GP	24 439	17·8 (17·3, 18·3)	24·3 (11·0, 39·3)
Consultant clinic	2768	11·7 (10·6, 13·3)	−1·6 (−53·7, 109·0)
Other	7298	13·9 (13·1, 14·7)	47·1 (21·3, 78·3)
Wales			
GI cancer			
Management			
Surgical specialty	9159	20·4 (19·6, 21·2)	26·0 (10·0, 44·3)
All other specialties	10 962	35·4 (34·5, 36·3)	28·6 (15·2, 43·4)
Admission source			
A&E	6632	29·1 (28·0, 30·2)	19·1 (5·2, 34·9)
GP	9848	31·0 (30·1, 32·0)	31·9 (16·3, 49·8)
Consultant clinic	1412	18·8 (16·8, 20·9)	40·5 (−46·0, 266·0)
Other	2229	22·3 (20·5, 24·0)	−18·8 (−42·0, 13·8)
Severe liver disease			
Management			
Consultant hepatologist or gastroenterologist	2620	16·0 (14·6, 17·5)	25·1 (−7·5, 69·1)
All other specialties	4853	18·9 (17·8, 20·0)	33·1 (9·8, 61·2)
Admitted to			
Liver transplant centre^‡^	–	–	–
All other transplant centres	7473	17·9 (17·0, 18·8)	32·0 (12·4, 55·1)
Admission source			
A&E	3700	19·8 (18·5, 21·1)	4·9 (−13·9, 27·9)
GP	3343	16·2 (14·9, 17·5)	65·0 (21·6, 124)
Consultant clinic	211	7·6 (4·4, 12·0)	§
Other	219	21·5 (16·2, 27·5)	131·0 (−84·3, 1149·0)

Values in parentheses are 95 per cent confidence intervals.

* These weekend mortality effect sizes are adjusted for patient age, sex and 11 major co-morbidities (for ICD-10 codes, see *Appendix S1*, supporting information), and are based on odds ratios from logistic regression modelling.

† Information on residential local authority was missing for 145 patients.

‡ There are no liver transplant centres in Wales. §Denotes no deaths after weekend admission. GI, gastrointestinal; A&E, accident and emergency department; GP, general practitioner.

When adjusting the weekend effects for additional factors, there was no significant impact on weekend effect sizes for year of admission, hospital size, day of death and surgical management (all less than 5 per cent reduction), or for social deprivation (less than 10 per cent reduction). After adjusting for admission source, the weekend effect fell from 26·2 (95 per cent c.i. 21·1 to 31·6) to 18·9 (14·0 to 24·1) per cent in England. No weekend effects were observed for patients admitted through consultant clinics, but there were significant weekend effects for all other sources of admission in England (*Table 4*).

#### Gastrointestinal cancer

There were large significant weekend effects on mortality for GI cancer overall in both countries (England: by 21·8 (95 per cent c.i. 19·1 to 24·5) per cent, *P* < 0·001; Wales: by 25·0 (15·0 to 35·9) per cent, *P* = 0·002). For each main GI cancer (oesophageal, gastric, colorectal, liver and pancreatic), there was a remarkably consistent and large weekend mortality effect in both populations of between 18·5 and 39·7 per cent. For gallbladder cancer, there was a similar but marginally non-significant weekend effect of 21·9 per cent in England, but an imprecise decreased weekend effect of 33·0 per cent in Wales (*Table 3*). After correction for multiple testing, the weekend effects were still significant for GI cancer overall and for the three most common GI cancers (colorectal, gastric and oesophageal) in England.

When assessing the impact of surgical involvement in the management of patients with cancer, mortality was much lower in those managed by surgeons compared with all other specialties (*Table 4*). The weekend mortality effect was also significantly higher in patients not managed by a surgeon in England (27·7 *versus* 14·0 per cent) and non-significantly higher in Wales (28·6 *versus* 26·0 per cent).

After adjusting for additional factors, there was no significant influence on weekend effect sizes for year of admission, hospital size, day of death and surgical management (all less than 5 per cent reduction), or for social deprivation (less than 10 per cent reduction). After adjusting for admission source, the weekend effect fell from 21·8 (95 per cent c.i. 19·1 to 24·5) to 15·4 (12·9 to 18·0) per cent in England. There was a significant inverse weekend effect for patients admitted with GI cancer via consultant clinics in England, but normal weekend effects for the other admission sources (*Table 4*).

### Relative survival

**Fig. 1:**
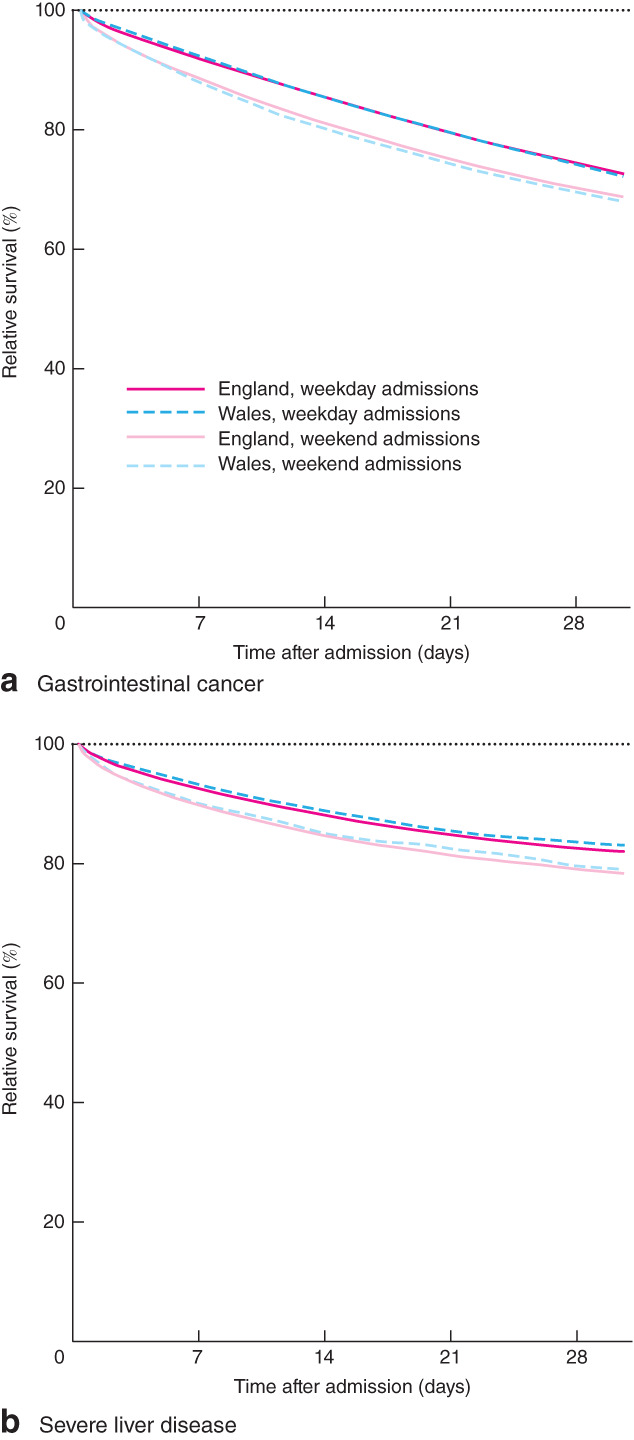
Relative survival up to 30 days after emergency admission for **a** gastrointestinal cancer and **b** severe liver disease (includes alcoholic liver disease and hepatic failure) on weekends and weekdays, compared with the general population of England and Wales. The black dotted line at 100 per cent denotes survival in the general population

For both GI cancer and severe liver disease, relative survival up to 30 days after admission, compared with the general population, was substantially worse for admissions at the weekend than on weekdays; most of this excess mortality for weekend admissions was evident by 7 days after hospitalization (*Fig. 1*).

## Discussion

The study reports on weekend mortality effects for major GI disorders. It covers medical and surgical GI disorders, severe liver disease and GI cancers, and, for confirmatory purposes, was based on two corresponding but independently collected information sources.

There was little or no evidence of strong weekend mortality effects for most general surgical and medical GI conditions. There was, however, some evidence of a weekend effect for upper GI bleeding and non-hiatal hernia, although there was no weekend effect for bleeds that were managed surgically. Weekend effect sizes of similar magnitude^15–18^, or larger^19^, have been reported previously for upper GI bleeding, although others^14,20,21^ have reported no weekend effect. This variable evidence for upper GI bleeding reflects, at least partly, variation across studies in study settings and case ascertainment criteria. There are no previous reports of a possible weekend effect for non-hiatal hernia.

Patients admitted at the weekend for general surgical or medical GI disorders were broadly comparable with those admitted on weekdays in terms of their median duration of inpatient stay, mean age and major co-morbidities, although it is possible that co-morbidities were documented less fully at weekends. However, for every disorder, admission rates in both populations were clearly and significantly lower at weekends than on weekdays, and weekend admissions were proportionately more often through accident and emergency departments than via general practitioners or consultant clinics. This indicates that there is a higher disease severity threshold for admission at the weekend, although in some hospitals this could be related to admission protocols, availability of services, beds or other factors. With fewer patients with mild disease admitted, a dilution effect during the week may partly explain the increased mortality following weekend admission.

The significantly increased mortality rate among patients admitted with severe liver disease at weekends (26·2 per cent in England and 32·0 per cent in Wales) has not been reported previously. Both mortality and the weekend effect were substantially less among patients managed by a consultant gastroenterologist or hepatologist. They were also lower among patients who were admitted to a liver transplant centre, although the reduction was confined largely to patients who were not local residents. This suggests that both overall mortality and the weekend effect are the result of the known lack of specialist hepatology resources in most centres^28,32^, which may have a greater impact at weekends. The lower mortality rate in patients admitted to a transplant centre from other areas also indicates some selection effect whereby the specialist centres tend not to accept transfers of patients with a poor prognosis. Strong weekend mortality effects were apparent for hepatic failure, alcoholic hepatic failure and alcoholic liver cirrhosis, but not for alcoholic hepatitis, a reversible disorder that carries lower mortality. Unscheduled admission rates of patients with severe liver disease were especially lower at weekends in both countries (43·3 per cent in England and 51·4 per cent in Wales), and admissions via general practitioners were greatly reduced at weekends, so that the dilution effect referred to above would apply. Most of the increased 30-day mortality for weekend admissions, compared with weekdays, occurred within 7 days of admission, which further suggests that more patients with end-stage disease are admitted at the weekend.

There was a remarkably consistent and large weekend mortality effect in both countries (of between 18·5 and 39·7 per cent) for each of the five major GI malignancies, oesophageal, gastric, colorectal, liver and pancreatic cancer. These findings are concordant with the limited previous evidence from Ontario, Canada, where weekend mortality effect sizes of 25 per cent for gastric cancer, 19 per cent for colonic cancer and 15 per cent for pancreatic cancer were reported^9^, and from New South Wales, Australia, with a weekend effect of 40 per cent for digestive cancers overall^12^.

Mortality rates and the weekend mortality effect were lower in patients with cancer who were managed by surgeons than by other specialties, and among patients admitted via a consultant clinic. The weekend effect was large for patients admitted by general practitioners; this is much less frequent at the weekend. A previous study^33^ of all major surgery in the north of England reported no weekend effect from day of admission, but a weekend effect from day of surgery. For each of the GI cancers, both unscheduled admission rates and the proportion of admissions via general practitioners were consistently lower at weekends than on weekdays, and most of the excess mortality for weekend admissions occurred soon after admission. This suggests that more patients with terminal disease are admitted at the weekend; this may be linked to reduced availability of hospice and end-of-life care in the community outwith normal working days^12,34^.

The major strengths of this study are that it provides evidence of possible weekend mortality effects for all major emergency GI disorders and, importantly for confirmatory purposes, is based on two independently collected information sources. It covers more than 2·2 million emergency GI admissions in England and over 150 000 in Wales, and is based on systematic validated record linkage methodology that has been used extensively in previous studies^3,15,22–24^. A further strength is that the GI disorders included in this study are defined by acute admissions and exclude elective admissions, which are often for investigation rather than treatment for active or present disease^35^. The inpatient data sources are confined to public hospitals, but these account for almost all of the acute admissions in the two study populations.

Study limitations are that the national administrative inpatient data used lack detailed information about disease history, severity and treatment, and the principal diagnosis in national administrative inpatient data is not accurate in all cases^36^. Furthermore, the diagnostic categories for the included GI disorders are limited by lack of granularity of ICD-10 coding. The coding of co-morbidities, although based on records of all secondary care received by patients currently and during the previous 5 years, is likely to be incomplete for some patients. The consultant specialty managing and treating patients was recorded in the administrative data only for the first and last episodes of the admission, and the specialties classified did not distinguish hepatology separately from gastroenterology. However, this would still have enabled ascertainment of almost all patients managed by surgical and gastroenterology specialties. The inpatient data sets do not include the time of admission, which has limited the ability to define the weekend more precisely than midnight on Friday to midnight on Sunday. In spite of these limitations, the sizes of the populations studied and the consistency of the findings across two neighbouring countries suggests validity.

## Supplementary Material

bjs10608-sup-0001-AppendixS1 **Appendix S1** ICD-10 codes used for the study of gastrointestinal disorders and patient co-morbidities (Word document)
