# Supplementary material for: Weekend admission and mortality for gastrointestinal disorders across England and Wales
Source: Br J Surg. 2017 Sep 19;104(12):1723–34. doi: 10.1002/bjs.10608 (PMC5656931; doi:10.1002/bjs.10608)
Supplement: bjs10608-sup-0001-AppendixS1 — Appendix S1 ICD-10 codes used for the study of gastrointestinal disorders and patient co-morbidities (Word document) [file bjs10608-sup-0001-appendixs1.docx]

**BJS10608**

**Weekend admission and mortality for gastrointestinal disorders across England and Wales**

**S. E. Roberts, T. H. Brown, K. Thorne, R. A. Lyons, A. Akbari, D. J. Napier, J. L. Brown and J. G. Williams**

**Appendix S1** ICD-10 codes used for the study of gastrointestinal disorders and patient co-morbidities

ICD-10 codes for GI disorders:

General surgical and medical GI disorders:

Upper GI bleeding (K22.6, K22.8, K25.0, K25.2, K25.4, K25.6, K26.0, K26.2, K26.4, K26.6, K27.0, K27.2, K27.4, K27.6, K28.0, K28.2, K28.4, K28.6, K29.0, I85.0, K92.0, K92.1 and - unless present with a secondary or subsidiary diagnosis of lower gastrointestinal disease - K92.2)

Perforated peptic ulcer and peritonitis (K25.1, K25.2, K25.5, K25.6, K26.1, K26.2, K26.5, K26.6, K27.1, K27.2, K27.5, K27.6, K65)

Gastritis (K29.0-K29.6)

Hernia (K40-K43, K45, K46)

Inflammatory bowel disease (K50 ,K51)

Non-infective gastroenteritis (K52)

Intestinal obstruction (K56)

Diverticular disease (K57)

Gallstone disease (K80, K81)

Acute pancreatitis (K85)

Intestinal infection (A00-A09)

Severe liver disease:

Alcoholic liver disease (K70) also differentiated according to:

- alcoholic hepatitis (K70.1)

- alcoholic cirrhosis of liver (K70.3)

- alcoholic liver failure (K70.4)

Hepatic failure (K72)

GI cancer (C15-C26):

Oesophageal cancer (C15)

Gastric cancer (C16)

Colorectal cancer (C18)

Liver cancer (C22)

Pancreatic cancer (C25)

Gallbladder cancer (C23)

ICD-10 codes for patient co-morbidities:

Ischaemic heart disease (120-I25)

Other cardiovascular diseases (I00-I15, I26-I52)

Cerebrovascular disease (I60-I69)

Other circulatory disease (I70-I99)

Malignancy (C00-C97)

Liver disease (K70-K77)

COPD (J40-J44)

Asthma (J45, J46)

Diabetes (E10-E14)

Renal failure (N17-N19)

Dementia (F00-F03, F05.1, G30)
